# miR-486-5p expression pattern in esophageal squamous cell carcinoma, gastric cancer and its prognostic value

**DOI:** 10.18632/oncotarget.7417

**Published:** 2016-02-16

**Authors:** Chuanli Ren, Hui Chen, Chongxu Han, Deyuan Fu, Lin Zhou, Guangfu Jin, Fuan Wang, Daxin Wang, Yong Chen, Li Ma, Xucai Zheng, Dongsheng Han

**Affiliations:** ^1^ Clinical Medical Testing Laboratory, Northern Jiangsu People's Hospital and Clinical Medical College of Yangzhou University, Yangzhou, China; ^2^ Geriatric Medicine, Northern Jiangsu People's Hospital and Clinical Medical College of Yangzhou University, Yangzhou, China; ^3^ Breast Oncology Surgery, Northern Jiangsu People's Hospital and Clinical Medical College of Yangzhou University, Yangzhou, China; ^4^ Department of Epidemiology and Biostatistics, Ministry of Education Key Laboratory of Modern Toxicology, School of Public Health, Nanjing Medical University, Nanjing, China; ^5^ Departments of Interventional Radiography, Northern Jiangsu People's Hospital and Clinical Medical College of Yangzhou University, Yangzhou, China; ^6^ Departments of Oncology, Northern Jiangsu People's Hospital and Clinical Medical College of Yangzhou University, Yangzhou, China; ^7^ Laboratory of Hematology, Northern Jiangsu People's Hospital and Clinical Medical College of Yangzhou University, Yangzhou, China; ^8^ Department of Head and Neck Surgery, Anhui Cancer Hospital, Hefei, China

**Keywords:** digestive system cancers, esophageal squamous cell carcinoma, gastric adenocarcinoma, micro RNA-486-5p, prognosis

## Abstract

Micro RNA (miR)-486-5p is often aberrantly expressed in human cancers. The aim of this study was to identify the prognostic value of miR-486-5p expression in digestive system cancers. Tissue microarrays were constructed with 680 samples including 185 esophageal squamous cell carcinomas (ESCCs), 90 gastric adenocarcinomas (GCs), and 60 digestive system cancer tissues from 10 ESCC, 10 GC, 10 colon, 10 rectum, 10 liver, 10 pancreatic cancer, and corresponding normal tissues. Twenty normal digestive system mucosa tissues from healthy volunteers were included as normal controls. In GC, miR-486-5p expression was decreased in 62.8% of cases (59/94), increased in 33.0% (31/94), and unchanged in 4.2% (4/94); in ESCC its expression was decreased in 66.2% (129/195), increased in 32.3% (63/195), and unchanged in 1.5% (3/195). Expression of miR-486-5p was decreased in 12, and increased in 8, of 20 cases of colon or rectum cancer; decreased in 6, and increased in 4, of 10 cases of liver cancer; and decreased in 8, and increased in 2, of 10 cases of pancreatic cancer. Multivariate and univariate regression analysis demonstrated that low/unchanged miR-486-5p predicted poor prognosis in ESCC (hazard ratio [HR], 4.32; 95% confidence interval [CI], 2.62–7.14; *P* < 0.001; HR, 3.88; 95% CI, 2.43–6.22; *P* < 0.001, respectively) and GC (HR, 2.46; 95% CI, 1.35–4.50; *P* = 0.003; HR, 2.55; 95% CI, 1.39–4.69; *P* = 0.002, respectively). MiR-486-5p might therefore be an independent tumor marker for evaluating prognosis in patients with ESCC or GC.

## INTRODUCTION

Incidence rates of esophageal cancer vary internationally by more than 21-fold; the highest rates are found in Eastern Asia and in Eastern and Southern Africa and the lowest rates are in Western Africa. Esophageal cancer is usually three to four times more common among men than women [1]. The two main types of esophageal cancer are squamous cell carcinoma and adenocarcinoma. From Northern Iran through Central Asia to North-Central China, 90% of cases are squamous cell carcinomas, compared with approximately 26% in the United States (among white individuals) [2, 3]. Patients are usually diagnosed with late-stage ESCC, which has limited treatment options. Therefore, there is a need for biomarkers to improve early detection and prognosis evaluation.

It is accepted that gastric carcinogenesis, like that of other malignancies, involves the accumulation of genetic and epigenetic changes [4]. However, the precise mechanisms underlying gastric carcinogenesis and prognosis remain unclear.

MicroRNAs (miRNAs) are noncoding RNA molecules of 18–25 nucleotides that inhibit translation or promote degradation of complementary messenger RNAs [5]. A single miRNA can influence the expression of hundreds of target genes, and miRNAs have been reported as key regulatory molecules in various diseases, including cancer [6]. Numerous studies have demonstrated that various miRNAs function as potential oncogenes or tumor suppressor genes during the progression of cancer [7], andmiRNAs have been associated with the carcinogenesis, metastasis, and prognosis of gastroesophageal cancers [8].

Aberrant expression of miR-486/miR-486-5p is associated with different types of disease. For example, high levels of circulating miR-486 are associated with acute myocardial infarction and may be used as a novel and powerful biomarker for acute myocardial infarction [9]. Reduced miR-486-5p expression is a common molecular event in human cancers [10–19] and miR-486 is frequently downregulated in hepatocellular carcinoma (HCC) tissues and cell lines. miR-486 inhibition in HCC cells enhances proliferation, invasion, and chemosensitivity to sorafenib [18]. miR-486 was shown to be significantly downregulated in primary gastric carcinoma (GC) and GC cell lines, and genomic loss of the miR-486 locus has been shown in approximately 25–30% of GCs, consistent with a tumor-suppressive role [15]. In contrast, miR-486-5p was upregulated in tissues of renal cell carcinoma. Deregulation of cancer-related miR-486-5p can also be a common event in both benign and malignant human breast tumors [17].

Early detection of lung adenocarcinoma in sputum using a panel of microRNA markers has been reported. Monitoring changes in plasma miR-486 expression might be an effective non-invasive method for predicting recurrence of early-stage non-small-cell lung cancer (NSCLC) [20]. Serum miR-486-5p holds great potential for further applications in the clinical diagnosis of lung cancers [21] and could be used to stratify patients with higher recurrence risk and potentially guide more effective surveillance tools for HCC [22]. We previously systematically screened the plasma miRNA profiles of patients with gastric non-cardia adenocarcinoma and healthy controls and identified miR-486-5p as one of five differentially expressed miRNAs [23].

However, there is an apparent contradiction between miR-486-5p expression in plasma and in GC tissues [23] and the clinical significance of miR-486-5p in GC remains unknown. We therefore investigated expression of miR-486-5p in tissues of different digestive system cancers including ESCC, GC, colon, rectum, liver, and pancreatic cancer, corresponding normal tissues, and normal digestive mucosa from healthy volunteers by miRNA locked nucleic acid (LNA) *in situ* hybridization, and evaluated its relationship with clinicopathologic parameters and prognosis.

## RESULTS

### Aberrant expression of miR-486-5p in digestive system cancers, paracancerous tissues, and normal mucosa of the digestive system

**Figure 1:**
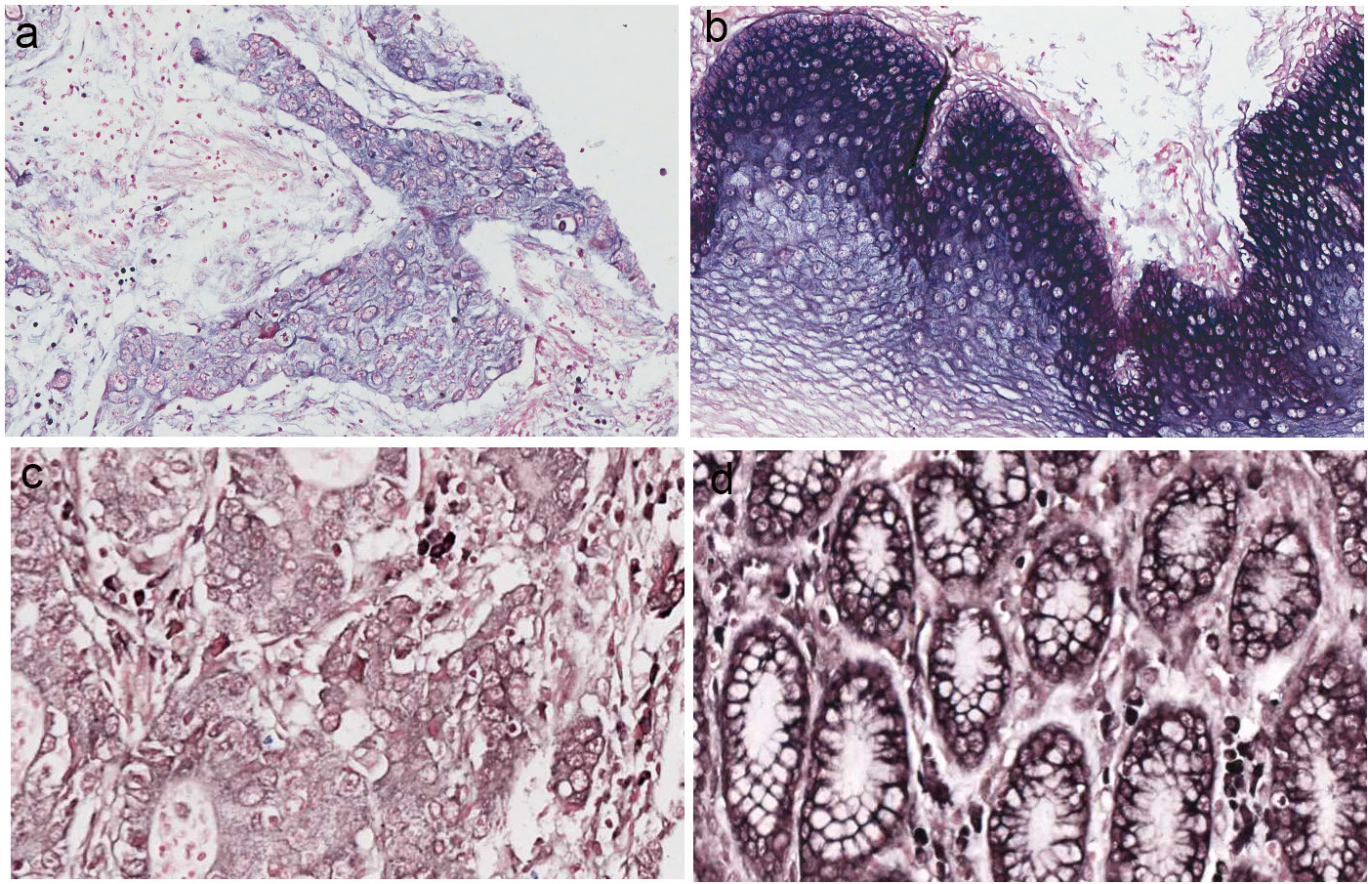
miRNA-486–5p levels were stained by *in situ* hybridization in esophageal squamous cell carcinomas (ESCC) and gastric carcinoma (GC) **a.** miR-486–5p expression in ESCC. **b.** miR-486–5p expression in ESCC neighboring normal tissue. **c.** miR-486–5p expression in GC. **d.** miR-486–5p expression in GC neighboring normal tissue.

The clinical data were list in Table 1 and Table 2. MiR-486-5p was mainly located in the cytoplasm of cells from digestive system cancers, neighboring normal tissue, and some samples of normal digestive mucosa (Figure 1). In GC, miR-486-5p expression was decreased in 62.8% (59/94), increased in 33.0% (31/94), and unchanged in 4.2% (4/94) of cases. In ESCC, its expression was decreased in 66.2% (129/195), increased in 32.3% (63/195), and unchanged in 1.5% (3/195). Expression of miR-486-5p was decreased in 60.0% (12/20) and increased in 40.0% (8/20) of colon or rectum cancers; decreased in 60.0% (6/10) and increased in 40.0% (4/10) of liver cancers; and decreased in 80.0% (8/10) and increased in 20.0% (2/10) of pancreatic cancers. Twenty normal esophageal, gastric, colon, rectum, liver, and pancreatic mucosa samples from healthy volunteers were included as normal controls. The expression of miR-486-5p was positive in 90.0% (18/20) and negative in 10.0% of normal digestive system mucosa samples from healthy volunteers. Aberrant miR-486-5p expression was thus detected in most digestive tissues, and its expression was decreased in the majority of cases of ESCC and GC, as well as other digestive system cancers (*P* < 0.01).

**Table 1:** Characteristics of the study subjects with esophageal squamous cell carcinoma

Clinicopathologic features	Number	Percentage (%)
**Age (years)**		
**<60**	56	28.7
**≥60**	139	71.3
**Gender**		
**male**	154	79.0
**female**	41	21.0
**Tumor size (cm)**		
**<5**	108	54.4
**≥5**	87	45.6
**Tumor site**		
**Upper**	3	1.5
**Upper-Middle**	157	80.5
**Low**	35	18.0
**Pathological type**		
**squamouscellcarcinoma**	194	99.5
**squamouscellcarcinoma combined with esophageal endocrine cell carcinoma**	1	0.5
**Tumor status**		
**T1+T2**	41	21.0
**T3+T4**	134	68.7
**Unknown**	20	10.3
**Nodal status**		
**negative**	45	23.1
**positive**	135	69.2
**unknown**	15	7.7
**Metastasis status**		
**M0**	184	94.4
**M1**	0	0
**Unknown**	11	5.6
**Tumor stage**		
**I**	8	4.3
**II**	86	46.5
**III**	85	45.9
**IV**	6	3.3
**unknown**	10	
**Follow-up time (months)**	45.6-93.6	
**Prognosis**		
**alive**	53	28.6
**dead**	132	71.4
**unknown**	10	
**patients lived for≥5 years**	34	18.4
**patients lived for<5 years**	145	78.4
**Unknown**	6	3.2

**Table 2:** Characteristics of the study subjects with gastric carcinoma

Clinicopathologic features	Number	Percentage (%)
**Age (years)**		
**<60**	32	34.0
**≥60**	62	66.0
**Gender**		
**male**	73	77.7
**female**	21	22.3
**Size(cm)**		
**<10**	22	23.4
**≥10**	72	76.6
**Tumor site**		
**cardia**	20	21.3
**Body**	18	19.1
**antrum**	38	40.4
**others**	18	19.2
**Pathological type adenocarcinoma**	94	100
**Nodal status**		
**negative**	27	28.7
**positive**	67	71.3
**Tumor stage**		
**I**	7	7.4
**II**	34	36.2
**III**	49	52.1
**IV**	4	4.3
**Follow up time (yeas)**	(6.6-7.3)	
**Prognosis**		
**alive**	24	25.5
**dead**	60	63.8
**patients lived for 5 years**	30	33.3
**unknown**	10	10.6

### Relationship between miR-486-5p expression and clinicopathologic features in ESCC and GC

There was a tendency towards a difference in TNM stage and local invasion between patients with low/unchanged versus high expression levels of miR-486-5p in ESCC and GC (χ^2^ = 3.047, *P* = 0.082; χ^2^ = 2.912, *P* = 0.088 respectively), but no significant correlations between miR-486-5p expression levels and other clinicopathologic variables, including age, sex, tumor site, TNM stage, tumor size, nodal status, distant metastasis, and depth of tumor invasion (all *P* > 0.05; Tables 3, 4).

**Table 3:** miR-486-5p expression and clinicopathologic features in patients with esophageal squamous cell carcinoma

Characteristics	MiR-486-5p low or unchanged (%)	MiR-486-5p high (%)	χ2	*P*-value
**Age(years)**			0.044	0.833
**<60**	30(26.1)	16(27.6)		
**≥60**	85(73.9)	42(72.4)		
**Gender**			3.090	0.079
**male**	96(83.5)	41(70.7)		
**female**	19(16.5)	17 (29.3)		
**Local invasion**			0.148	0.7
**T1+T2**	23 (21.9)	14 (24.6)		
**T3+T4**	82 (78.1)	43 (75.4)		
**TNM stage**			3.047	0.082
**I + II**	52 (47.7)	37 (61.7)		
**III + IV**	57 (52.3)	23 (38.3)		
**Nodal status**			1.049	0.306
**positive**	60(53.1)	26(44.8)		
**negative**	53(46.9)	32(55.2)		
**Tumor size(cm)**			0.033	0.856
**≥5**	47(44.3)	24(42.9)		
**<5**	59(55.7)	32(57.1)		

**Table 4:** miR-486-5p expression and clinicopathologic features in patients with gastric carcinoma

Characteristics	miR-486-5p Low/unchanged expression (n=57)	miR-486-5p High expression (n=27)	Significance	
			χ2 or Fisher's exact test	*P*-value
**Age(years)**			4.673	0.031
**<60**	14(24.6)	13(48.1)		
**≥60**	43(75.4)	14(51.9)		
**Gender**			1.117	0.291
**Male**	46(80.7)	19(70.4)		
**Female**	11(19.3)	8(29.6)		
**Local invasion**			2.912	0.088
**T1+T2**	5 (8.8)	6 (22.2)		
**T3+T4**	52 (91.2)	21 (77.8)		
**Site**			1.625	0.202
**gastric cardia**	13 (22.8)	3(11.1)		
**non-cardia**	44 (77.2)	24(88.9)		
**TNM stage**			0.455	0.5
**I + II**	23 (40.3)	13 (48.1)		
**III + IV**	34 (59.7)	14 (51.9)		
**Nodal status**			2.422	0.120
**positive**	45(78.9)	17(63.0)		
**negative**	12(21.1)	10(37.0)		
**Distant metastasis**				0.591
**M0**	55(96.5)	25(92.6)		
**M1**	2(3.5)	2(7.4)		
**Tumor size (cm)**				1.0
**≥10**	9(15.8)	4(14.8)		
**<10**	48(84.2)	23(85.2)		
**Depth of tumor invasion Mucosa, submucosa, muscularis propria, subserosa Penetration of serosa, adjacent structures**	40(70.2)	20(74.1)		0.712
	17(29.8)	7(25.9)	0.136	

### Survival analysis

The median overall survival (OS) in the study cohorts for ESCC and GC was 19.5 and 38 months and the longest OS was 93.6 and 87.6 months, respectively (Tables 1, 2). Kaplan–Meier analysis demonstrated that low or unchanged expression of miR-486-5p, stage of disease, tumor status, and node status were significant negative prognostic predictors for OS in patients with ESCC (*P* < 0.001, *P* < 0.001, *P* = 0.001, *P* < 0.001, respectively) and those with GC (*P =* 0.002, *P* < 0.001, *P* = 0.001, *P* = 0.005, respectively). Other clinicopathologic characteristics, including age, sex, and tumor size and location, were not significantly associated with prognosis in ESCC or GC (*P >* 0.05; Tables 5, 6).

**Table 5:** Univariate analysis of survival in esophageal squamous cell carcinoma

Variable	Mean survival time Month(±SE)	95% CI(Month)	P
**Age (years)**			0.573
**<60**	39.5(4.9)	29.9 −49.0	
**≥60**	38.2(3.2)	31.9 −44.6	
**Gender**			0.483
**Male**	40.0(3.1)	24.0-46.1	
**Female**	24.7(3.3)	18.2-31.1	
**Pathological grade**			0.978
**I–II**	39.2(3.2)	33.0-45.4	
**III**	37.1(5.2)	26.8-47.3	
**Stage of disease**			0.000
**I–II**	50.7(4.0)	42.8-58.6	
**III –IV**	22.6 (2.8)	17.2-28.0	
**Tumor status (p)**			0.001
**T1-T2**	47.7(5.1)	37.7-57.6	
**T3-T4**	32.7 (3.0)	26.9-38.5	
**Node status**			0.000
**Negative**	49.4 (4.1)	41.4-57.5	
**Positive**	26.9 (3.0)	21.1-32.8	
**MiR-486-5p**			0.000
**High expression**	59.5(4.4)	50.8-68.2	
**Low/unchanged**	27.8 (2.7)	22.4-33.3	
**Tumor size (cm)**			
**≥5**	37.2 (4.1)	18.5-38.2	0.286
**<5**	40.4 (3.6)	29.2-45.3	

**Table 6:** Univariate analysis of survival in gastric carcinoma

Variable	Mean survival time Month (±SE)	95% CI(Month)	P
**Age (years)**			0.689
**<60**	46.5(6.2)	34.4-58.6	
**≥60**	43.7(4.1)	35.8-51.7	
**Gender**			
**Male**	44.7(3.9)	37.1-52.4	0.955
**Female**	44.8(7.0)	31.2-58.4	
**Tumor site**			0.365
**Gastric cardia**	38.3 (6.2)	26.2-50.4	
**Non-cardia**	46.2 (3.9)	38.5-53.9	
**Stage of disease**			<0.001
**I–II**	58.5(5.2)	48.2-68.8	
**III –IV**	34.4 (3.9)	26.8-42.1	
**Tumor status (p)**			0.001
**T1-T2**	76.1 (8.1)	60.3-91.9	
**T3-T4**	39.9 (3.4)	33.3-46.5	
**Node status**			0.005
**Negative**	61.3 (6.7)	48.1-74.5	
**Positive**	38.9 (3.7)	31.7-46.2	
**Distant metastasis**			0.120
**No**	45.8 (3.5)	38.9-52.8	
**Yes**	25.0 (6.6)	12.0-38.0	
**MiR-486-5p**			0.002
**High expression**	61.9 (5.2)	51.6-72.2	
**Low/unchanged**	36.7 (3.9)	29.0-44.5	
**Tumor size (cm)**			0.034
**≥10**	30.4 (6.7)	17.3-43.6	
**<10**	47.7(3.8)	40.3-55.1	

**Figure 2:**
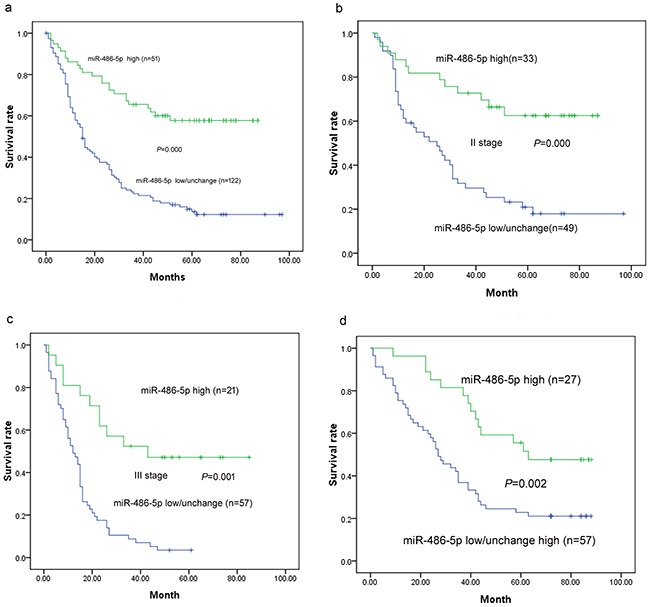
Survival curves in patients with ESCC and GC according to miRNA-486-5p levels **a.** Overall survival curves in patients with ESCC according to miRNA-486-5p levels (*P* < 0.001). **b.** Survival curves in stage II ESCC according to miRNA-486-5p levels (*P* < 0.001). **c.** Survival curves in stage III GC according to miRNA-486-5p levels (*P* = 0.001). **d.** Survival curves in patients with GC according to miRNA-486-5p levels (*P* = 0.002).

The prognosis of ESCC patients with low/unchanged miR-486-5p expression was significantly poorer than that of ESCC patients with high miR-486-5p expression (*P* < 0.001; Figure 2). The mean survival times were 59.5 months for high miR-486-5p expression and 27.8 months for low/unchanged miR-486-5p expression. After stratification of patients according to American Joint Committee on Cancer stage, low/unchanged miR-486-5p expression remained a significant predictor of poor survival in stage II (34.8 vs. 63.2 months; *P* < 0.001, *n* = 82) and stage III (15.2 vs. 50.0 months; *P* < 0.001, *n* = 78) ESCC. Variables that were significantly associated with OS in univariate analysis were included in Cox proportional hazards, multivariate regression analysis.

The prognosis of GC patients with low/unchanged miR-486-5p expression was significantly poorer than that of GC patients with high miR-486-5p expression (*P* < 0.001; Figure 2). The mean survival times were 61.9 months for high miR-486-5p expression and 36.7 months for low/unchanged miR-486-5p expression.

In univariate analysis for ESCC, tumor status (hazard ratio [HR], 2.12; 95% confidence interval [CI], 1.31–3.43; *P* = 0.002), stage (HR, 2.24; 95% CI, 1.57–3.19; *P* < 0.001), lymph node metastasis (HR, 1.94; 95% CI, 1.37–2.76; *P* < 0.001), low/unchanged miR-486-5p (HR, 4.32; 95% CI, 2.62–7.14; *P* < 0.001), and tumor size (HR, 1.92; 95% CI, 1.18–3.12; *P* = 0.009) predicted poor prognosis. Age (HR, 1.098; 95% CI, 0.71–1.70; *P* = 0. 675), sex (HR, 0.91; 95% CI, 0.58–1.43; *P* = 0.688), and pathologic grade (HR, 0.876; 95% CI, 0.56–1.37; *P* = 0.562) were not correlated to prognosis (*P >* 0.05) (Table 7). Moreover, in multivariate analysis for ESCC, tumor status (HR, 1.89; 95% CI, 1.12–3.19; *P* = 0.018), stage (HR, 2.46; 95% CI, 1.66–3.65; P < 0.001), lymph node metastasis (HR, 1.87; 95% CI, 1.26–2.78; *P* < 0.001), and low/unchanged miR-486-5p (HR, 3.88; 95% CI, 2.43–6.22; *P* < 0.001) predicted poor prognosis (Table 7).

**Table 7:** Multivariate Cox regression analysis of potential prognostic factors for survival in patients with esophageal squamous cell carcinoma

Variables	Univariate analysis		Multivariate analysis	
	HR(95%CI)	*P*-value	HR(95%CI)	*P*-value
**Tumor status, T3-T4 vs. T1-T2**	7.23(1.76-29.72)	0.006	0.243(0.056-1.049)	0.058
**Stage, III–IV vs. I–II**	2.64(1.51-4.62)	0.001	0.367(0.209-0.644)	0.000
**LNM, yes vs. no**	2.53(1.28-5.02)	0.008		0.472
**Low 486-5p vs. High 486-5p**	2.46(1.35-4.50)	0.003	2.55(1.39- 4.69)	0.002
**Tumor size (cm), ≥10 vs. <10**	1.97(1.04-3.76)	0.039	0.479(0.247-0.932)	0.03
**Age (years), ≥60 vs.<60**	1.12(0.64–1.95)	0.692		0.488
**Gender, male vs. female**	1.02(0.55–1.88)	0.956		0.956
**Pathological grade, III vs. I II**	1.48(0.80–2.75)	0.212		0.621

In univariate analysis for GC, tumor status (HR, 7.23; 95% CI, 1.76–29.72; *P* = 0.006), stage (HR, 2.64; 95% CI, 1.51–4.62; *P* = 0.001), lymph node metastasis (HR, 2.53; 95% CI, 1.28–5.02; *P* = 0.008), low/unchanged miR-486-5p (HR, 2.46; 95% CI, 1.35–4.50; *P* = 0.003), and tumor size (HR, 1.97; 95% CI, 1.04–3.76; *P* = 0.039) predicted poor prognosis. Age (HR, 1.12; 95% CI, 0.64–1.95; *P* = 0. 692) and sex (HR, 1.02; 95% CI, 00.55–1.88; *P* = 0.956) were not correlated to prognosis (P > 0.05) (Table 8). Moreover, in multivariate analysis for GC, stage (HR, 2.72; 95% CI, 1.55–4.78; *P* < 0.001), low/unchanged miR-486-5p (HR, 2.55; 95% CI, 1.39–4.69; *P* = 0.002), and tumor size (HR, 2.09; 95% CI, 1.07–4.05; *P* = 0.03) predicted poor prognosis (Table 8).

**Table 8:** Multivariate Cox regression analysis of potential prognostic factors for survival in patients with gastric carcinoma

Variables	Univariate analysis		Multivariate analysis	
	HR(95%CI)	*P*-value	HR(95%CI)	*P*-value
**Tumor status, T3-T4 vs. T1-T2**	7.23(1.76-29.72)	0.006	0.243(0.056-1.049)	0.058
**Stage, III–IV vs. I–II**	2.64(1.51-4.62)	0.001	0.367(0.209-0.644)	0.000
**LNM, yes vs. no**	2.53(1.28-5.02)	0.008		0.472
**Low 486-5p vs. High 486-5p**	2.46(1.35-4.50)	0.003	2.55(1.39- 4.69)	0.002
**Tumor size (cm), ≥10 vs. <10**	1.97(1.04-3.76)	0.039	0.479(0.247-0.932)	0.03
**Age (years), ≥60 vs.<60**	1.12(0.64–1.95)	0.692		0.488
**Gender, male vs. female**	1.02(0.55–1.88)	0.956		0.956
**Pathological grade, III vs. I II**	1.48(0.80–2.75)	0.212		0.621

Abbreviations: *HR* hazard ratio, *CI* confidence interval, *LNM* lymph node metastasis

*P < 0.05, statistically significant

## DISCUSSION

To date, the antiapoptotic genes *OLFM4* [15], *SIRT1* [24], *PIM-1* [25], and *DOCK3* [26], the tumor suppressor *PTEN* [27, 28], the cell proliferation and invasion genes *CITRON* and *CLDN10* [18], and *FGF9* [29] expressed by cancer-associated fibroblasts have been reported as potential targets for miR-486/miR-486-5p in malignant disease. However, the mechanistic role of miR-486-5p as either an oncogene or tumor suppressor in digestive system cancers remains largely unknown.

MiR-486-5p was also shown to directly inhibit *PIK3R1* expression in gastric cancer cells [30]. In our previous study, we found that miR-486-5p inhibits *FGF9* and that high expression of miR-486-5p in GC is associated with shorter overall survival [29]. In addition to increased levels of circulating miR-486-5p in the plasma of patients with GC or lung cancer in our previous study [23, 31], levels of miR-486-5p in saliva and saliva supernatant are significantly upregulated and show great promise as biomarkers for the detection of esophageal cancer [32]. In a previous study, increased plasma levels of miR-21 tended to be associated with greater vascular invasion (*P* = 0.1554) and showed a high correlation with recurrence (*P* = 0.0164) in ESCC [33]. However, the expression and prognostic value of miR-486-5p in patients with resected ESCC is unknown.

In our current study, the expression of miR-486-5p was increased in 90.0% (18/20) of esophageal, gastric, colon, rectum, liver, and pancreatic mucosa from healthy volunteers and decreased in 10.0% of normal digestive system mucosa samples. MiR-486-5p may have a protective function in healthy volunteers, as supported by a study suggesting that miR-486-5p inhibits pulmonary fibrosis [34]. We compared miR-486-5p expression levels in ESCC and paracancerous tissues by miRNA-LNA *in situ* hybridization and showed that miR-486-5p expression was significantly decreased in most, but not all, ESCC tissues—expression was decreased in 66.2% (129/195), increased in 32.3% (63/195), and unchanged in 1.5% (3/195) of ESCC tissues compared with paracancerous normal tissue. This suggests that miR-486-5p expression might have different prognostic implications in different individuals. MiR-486-5p was previously found to be markedly overexpressed in most primary glioma tissues compared with paired tumor-adjacent brain tissues, which significantly correlated with NF-kappaB activation status [35].

To the best of our knowledge, this study is the first to systematically explore the expression of miR-486-5p and its association with clinicopathologic features and prognosis of ESCC, GC, and other types of digestive system cancers, with a view to identifying curative therapies. Navon and colleagues recently reported that miR-486 was the top ranking underexpressed miRNA in cancer based on minRCoS ranking [12]. However, we found overexpression of miR-486-5p in 32.3% (63/195) of ESCC, 33.0% (31/94) of GC, 40.0% (8/20) of colon or rectum cancer, 40.0% (4/10) of liver cancer, and 20.0% (2/10) of pancreatic cancer patients. This implies heterogeneous expression of miR-486-5p, with high expression in a fraction of digestive system cancers. Moreover, we showed for the first time that miR-486-5p expression is increased in most esophageal, gastric, colon, rectum, liver, and pancreatic mucosal tissues from healthy volunteers. A previous four-phase study detected increased miR-486-5p expression during the early stage of GC and identified five plasma miRNAs (miR-16, miR-25, miR-92a, miR-451, and miR-486-5p) that might serve as potential diagnostic markers for early-stage gastric non-cardia adenocarcinoma [23]. Our research group previously found that miR-486-5p, as one member of the four-miRNA signature from the serum, may serve as a noninvasive predictor for the overall survival of NSCLC [31]. In the present study, ESCC and GC patients with higher miR-486-5p expression showed better prognoses than those with lower or unchanged expression. Moreover, univariate and multivariate Cox regression analysis of potential prognostic factors for survival identified a significant relationship between low/unchanged miR-486-5p expression and poor prognosis of ESCC (*P* < 0.001, *P* < 0.001 respectively) and GC (*P* = 0.002, *P* = 0.003 respectively). These results are in accordance with our previous finding that high expression of miR-486-5p in gastric cancer implied better prognosis [29]. Therefore, the detection of miR-486-5p in the circulation or tissues may provide new information to help in early diagnosis and prognostic evaluation of digestive system cancers, even though the molecular function of miR-486-5p in digestive system cancers remains unknown.

Several possible explanations for the observed downregulation of miR-486-5p in tumor tissues have been reported. For example, miR-486-5p is located on chromosome 8p11.21, which is one of the most frequently deleted genomic regions containing potential tumor suppressor genes, in various types of tumors including GC and lung cancer [15, 36]. In our previous work, we found FGF9 is one of potential target genes of miR-486-5p in GC. FGF9 expression was decreased in 69.0% (58/84) of GC samples compared with normal paracancerous tissues using immunohistochemical analysis[29].

The results of this study indicate that digestive system cancers, especially cases of ESCC and GC with low or unchanged miR-486-5p expression compared with neighboring normal tissues, have a relatively poor prognosis, whereas high miR-486-5p expression is associated with a better prognosis. Although further studies are needed to confirm this work, these results suggest that miR-486-5p may represent a valuable independent prognostic indicator, as well as a potential target for the treatment of digestive system cancers.

## MATERIALS AND METHODS

### Patients and tissue samples

Four biochips were used to detect miR-486-5p in digestive system cancers, especially ESCC, and neighboring normal tissues, and normal digestive normal mucosa were also included as controls. A total of 680 digestive cancer tissues, neighboring normal tissues, and normal digestive normal mucosa were included in the study. Samples from tumor tissue, corresponding neighboring normal tissue, and complete normal mucosa were collected from patients with histologically diagnosed digestive cancers who underwent surgical resection between June 2006 and December 2010. Data for 12 patients were excluded because the corresponding dots were not on the chips during this experiment or because of loss to follow-up. Paraffin-embedded tissue samples were collected retrospectively from archival material stored in the Biobank Center at the National Engineering Center for Biochip at Shanghai (Shanghai Outdo Biotech Cop., Ltd).

First, miR-486-5p expression was preliminarily detected in 140 dots on the digestive system chip. Second, miR-486-5p was independently validated in 360 dots for ESCC and neighboring normal tissues. Third, the clinical and prognostic value of miR-486-5p expression was evaluated for ESCC.

The following clinicopathologic data were obtained from the original pathology reports: age, sex, tumor size, location and invasion, lymph node metastases, grade of differentiation, and tumor stage. Staging of GC was assessed according to American Joint Committee on Cancer criteria. The clinical and pathologic data for the patients are provided in Table 1. Written informed consent was obtained from all patients, and the protocol was approved by the Ethical Committee of the National Engineering Center for Biochip at Shanghai.

Follow-up times were measured from the date of surgery to the date of death for all 185 ESCC patients. Data for 7 ESCC and 6 GC patients were excluded from miR-486-5p analysis because the dots were not included in the chips used in this experiment. The last follow-up point was in September 2014. The median follow-up time was 5.9 years (range 3.8–7.8 years). Among the patients, 132 died during the follow-up period.

### Tissue microarray construction

Tissue microarrays were constructed using appropriate tissue cores from formalin-fixed and paraffin-embedded samples as described previously [37]. Briefly, appropriate tumor areas and corresponding non-tumor gastric samples were selected by pathologists and a single core (diameter 0.6 mm) was taken from each tissue. Tissue microarray blocks were constructed using an automated tissue arrayer (Beecher Instruments, Sun Prairie, WI, USA). The array blocks were cut into 5-μm sections, and the sections were stained with hematoxylin and eosin to verify the presence of tumor cells. In all cases, tissue cores obtained from normal adjacent tissue served as internal controls.

### miRNA-LNA *in situ* hybridization

miRNA-LNA *in situ* hybridization was performed using antisense oligonucleotide probes for miR-486-5p (Exiqon Inc., Woburn, MA, USA) with Scramble-miR as a negative control. A solution of 20 nM probe in hybridization buffer was applied to the array slides, which were covered with Nescofilm (Bando Chemical Co., Kombe, Japan) and overnight at 55°C in a humidified chamber. Hybridized probes were detected by incubation with anti-digoxigenin–alkaline phosphatase conjugate at 37°C for 30 min, followed by nitro blue tetrazolium/5-bromo-4-chloro-3-indolyl-phosphate to develop a blue color. Finally, the cells were counterstained with nuclear fast red for 3–5 min and mounted on slides. Sections were scored as follows: 0, ≤5% labeled cells; 1, 5–30% labeled cells; 2, 31–70% labeled cells; 3, ≥71% labeled cells. The staining intensity was scored similarly, with 0 indicating negative staining, 1 weakly positive, 2 moderately positive, and 3 strongly positive. The scores for the percentage of positive tumor cells and staining intensity were summed to generate an immunoreactive score for each specimen. A final score of 0–1 indicated negative expression (−), 2–3 indicated weak expression (+), 4–5 indicated moderate expression (++), and 6 indicated strong expression (+++). Each sample was examined separately and scored by two pathologists [38]. In this analysis, differences in the expression level of miR-486-5p in ESCC tissues are expressed relative to the level of miR-486-5p in normal paracancerous tissues. In our research, increased levels of miR-486–5p in GC samples indicate that the final score of miR-486–5p expression in GC is higher than that of normal paracancerous tissues. Decreased levels of miR-486–5p in GC indicate that thefinal score of miR-486–5p expression in GC samples is higher than that of normal paracanceroustissues. Unchanged levels of miR-486–5p in GC samples indicate that the final score ofmiR-486–5p expression in GC samples is equal to that of normal paracancerous tissues.

And the expression level of miR-486-5p in normal mucosal samples from healthy volunteers are used as normal internal controls. The expression of miR-486-5p in normal mucosal samples were judged as positive or negative by the staining status.

### Statistical analysis

Associations between clinicopathologic parameters and miR-486-5p expression were analyzed using χ^2^-tests. When sample numbers in some categorical cells were lower than 5, the Fisher's exact test was employed. Overall survival was calculated and survival curves were plotted using the Kaplan–Meier method; differences between groups were compared using log-rank tests. Significant variables in univariate models were further analyzed by multivariate Cox proportional hazards regression models to identify independent prognostic factors. All analyses were performed using the SPSS software package (SPSS Inc., Chicago, IL, USA, version 17.0). All tests were two-sided and *P* values < 0.05 were considered statistically significant.
